# The Impact of Evidence Reliability on Sensitivity and Bias in Decision Confidence

**DOI:** 10.1037/xhp0000404

**Published:** 2017-04-06

**Authors:** Annika Boldt, Vincent de Gardelle, Nick Yeung

**Affiliations:** 1 Department of Experimental Psychology, University of Oxford, and Department of Psychology, University of Cambridge; 2 Paris School of Economics and CNRS, Paris, France; 3 Department of Experimental Psychology, University of Oxford

**Keywords:** decision making, confidence, metacognition, perceptual averaging, over- and underconfidence

## Abstract

Human observers effortlessly and accurately judge their probability of being correct in their decisions, suggesting that metacognitive evaluation is an integral part of decision making. It remains a challenge for most models of confidence, however, to explain how metacognitive judgments are formed and which internal signals influence them. While the decision-making literature has suggested that confidence is based on privileged access to the evidence that gives rise to the decision itself, other lines of research on confidence have commonly taken the view of a multicue model of confidence. The present study aims at manipulating one such cue: the perceived reliability of evidence supporting an initial decision. Participants made a categorical judgment of the average color of an array of eight colored shapes, for which we critically manipulated both the distance of the mean color from the category boundary (evidence strength) and the variability of colors across the eight shapes (evidence reliability). Our results indicate that evidence reliability has a stronger impact on confidence than evidence strength. Specifically, we found that evidence reliability affects *metacognitive readout*, the mapping from subjectively experienced certainty to expressed confidence, allowing participants to adequately adjust their confidence ratings to match changes in objective task performance across conditions.

## Public Significance Statement

People constantly face various types of decisions, which are commonly accompanied by an inherent feeling of (in)correctness: Just as realizing the tennis ball we played will most likely hit the net, we can feel more or less confident regarding our recent car purchase. People’s confidence judgments have been found to be surprisingly accurate. However, little is known about the underlying mechanisms that give rise to them. In this study, we suggest that variability in the information we receive from the outside world is of particular importance for how confident we feel in our decisions—more so than the extent to which evidence favors one over another choice option. Specifically, we find that this variability affects how people translate their internal feelings of certainty into the confidence judgments they express.

When we make decisions we experience a subjective sense of confidence that varies from certainty that we chose correctly to feelings that we were guessing or even made a mistake. These confidence judgments play a crucial role in adaptive decision making, for example in guiding search for further information that might better inform the decision (Bonaccio & Dalal, 2006) and in integrating diverging opinions in group decisions (Bahrami et al., 2010). In experiments using simple perceptual comparisons, subjects readily estimate the likelihood that a just-given response was correct, whether using a verbal scale (e.g., Baranski & Petrusic, 1998; Boldt & Yeung, 2015; Lebreton, Abitbol, Daunizeau, & Pessiglione, 2015), a numerical one (Baranski & Petrusic, 1994; Gigerenzer, Hoffrage, & Kleinbölting, 1991; Merkle & Van Zandt, 2006), or a confidence comparison procedure (e.g., de Gardelle & Mamassian, 2015). These subjective judgments typically correlate strongly with objective task performance, therefore reflecting true metacognitive insight into participants’ own mental processes. There is growing interest in the computational and neural basis of these confidence judgments, and substantial progress has been made through linking theories of confidence to established models of the decision process itself such as signal detection theory and evidence accumulation models (Kiani, Corthell, & Shadlen, 2014; Moreno-Bote, 2010; Yeung & Summerfield, 2012, 2014; Zylberberg, Roelfsema, & Sigman, 2014).

The primary focus of past research has been on characterizing the *sensitivity* (sometimes labeled *resolution*) of confidence judgments—that is, the degree to which people can distinguish their correct and incorrect responses such that greater subjective confidence predicts greater objective accuracy. Sensitivity has been shown to vary systematically with factors such as task difficulty, time pressure for both the initial decision and later confidence judgment, and the consensuality of decisions across individuals: Confidence better predicts accuracy when the task is easy (Baranski & Petrusic, 1994), when the initial decision is made under time pressure but when confidence judgments are made at leisure (Moran, Teodorescu, & Usher, 2015; Pleskac & Busemeyer, 2010), and when there is collective agreement that the choice made is the correct one (Koriat, 2012). Many of these core findings can be explained using formal decision frameworks such as a race model in which evidence counters for each possible decision race toward a threshold value. Here, for instance, confidence can be quantified as the balance of evidence between counters at the time of the decision (Vickers & Packer, 1982): The balance of evidence will on average be larger for easy decisions in which the available evidence strongly favors one choice over the alternatives (cf. Baranski & Petrusic, 1994) and will grow if more time is allowed for the confidence judgment to be made (cf. Moran et al., 2015; Pleskac & Busemeyer, 2010). Recent single-unit recording studies have identified possible neural correlates of such a balance-of-evidence signal in rats (Kepecs, Uchida, Zariwala, & Mainen, 2008) and monkeys (Kiani & Shadlen, 2009; Komura, Nikkuni, Hirashima, Uetake, & Miyamoto, 2013). According to this perspective, confidence is a direct readout of the decision process—a function of the evidence that drives our choices.

This view diverges from the prevailing view in another prominent line of research—on metacognitive processes in memory—where it is widely assumed that judgments do not rely on direct readout of memory strength, but instead depend on multiple heuristic cues (Koriat & Levy-Sadot, 2001; Schwartz, 1994) such as the familiarity of the question (Reder & Ritter, 1992) and the accessibility of information at retrieval (Koriat, 1993). Interestingly, some findings within the decision-making literature suggest that cues or heuristics external to the decision process may likewise influence people’s confidence in their decisions, including decision speed (Audley, 1960; Kiani et al., 2014; Zylberberg, Barttfeld, & Sigman, 2012) and the familiarity of the decision options (De Martino, Fleming, Garrett, & Dolan, 2013). Other findings suggesting that confidence and objective accuracy are dissociable include, for example, a recent study by Fleming et al. (2015) in which stimulation of the motor cortex led to disruptions of confidence but not objective accuracy in a perceptual decision-making task. Conversely, Rahnev, Nee, Riddle, Larson, and D’Esposito (2016) reported that stimulation to the anterior prefrontal cortex results in improved metacognitive insight, while not changing overall task performance.

Here, we build on these findings that confidence and accuracy are dissociable to test the hypothesis that confidence is critically influenced by the perceived reliability of the evidence on which decisions are based. This hypothesis derives from recent theoretical work (Yeung & Summerfield, 2014; see also Meyniel, Sigman, & Mainen, 2015; Pouget, Drugowitsch, & Kepecs, 2016) linking confidence judgments to the hypothesis that perceptual uncertainty is encoded as the variance in firing rate across a neural population, with increased uncertainty leading to down-weighting of that evidence source in perceptual integration (Beck et al., 2008; Ma, Beck, Latham, & Pouget, 2006). Extending these ideas to the decision-making process, it has been proposed that variability in stimulus representations may provide a crucial cue to confidence, with the specific prediction that increased evidence variance—that is, decreased reliability—should result in reduced confidence (Meyniel et al., 2015; Yeung & Summerfield, 2014). According to this Bayesian account, confidence reflects the precision of a posterior distribution: The less precise the evidence (i.e., the wider the posterior probability distribution), the less confident should be any decision based on this evidence (Yeung & Summerfield, 2014). However, experimental evidence on this point has been contradictory to date with some studies suggesting that confidence increases with evidence variability (Zylberberg et al., 2014; Zylberberg, Fetsch, & Shadlen, 2016), and other studies suggesting the opposite pattern (e.g., Allen, Frank, Schwarzkopf, Fardo, Winston, Hauser, & Rees, 2016; Irwin, Smith, & Mayfield, 1956; Spence, Dux, & Arnold, 2016). A recent study by Spence et al. (2016) carefully matched the effects of evidence reliability and evidence strength on performance, and convincingly demonstrated effects of evidence reliability over and above the effect of evidence mean on confidence. However, another study with a corresponding approach reported less consistent effects (de Gardelle & Mamassian, 2015). Here, we adopted this approach of directly contrasting the effects of evidence mean on confidence, but used factorial crossing of these two manipulations to enable us to isolate changes in confidence from changes in first-order performance, while also carefully controlling for decision speed as well as decision accuracy.

In this way, the present study investigated the impact of evidence reliability on confidence judgments, and crucially did so not only in the context of its effect on metacognitive sensitivity—that is, on people’s ability to discriminate their correct responses from their errors—but also on a second crucial feature of confidence: the degree to which judgments are accurately calibrated versus exhibiting systematic bias toward under- or overconfidence, independent of objective accuracy. This is an important but much less understood aspect of confidence. Confidence bias may vary across individuals as a stable trait—for example, in an education setting, one student might habitually express her opinions with confidence regardless of their veracity (overconfidence, or high bias), whereas another may express carefully thought-through ideas with unwarranted caution (underconfidence, or low bias). Biases may also vary within an individual according to the situation—for example, a student might voice the same opinion with high confidence when talking to a trusted peer (high bias) but hesitantly in a crowded classroom with an intimidating professor (low bias). Conceptually, then, biases can emerge in mapping from subjectively experienced certainty to expressed confidence, for example, in terms of the verbal or numerical labels that are required by an experimenter (Overgaard & Sandberg, 2012) or elicited in group interactions (Fusaroli et al., 2012). Henceforth, we refer to this mapping as the process of *metacognitive readout*.

In existing empirical studies, overconfidence has consistently been observed when the task is difficult, whereas calibrated or even underconfident evaluations are observed when the task is easy—the *hard-easy effect* (Baranski & Petrusic, 1994; Drugowitsch, Moreno-Bote, & Pouget, 2014; Gigerenzer et al., 1991; Lichtenstein & Fischhoff, 1977; Merkle & Van Zandt, 2006; Pleskac & Busemeyer, 2010; Suantak, Bolger, & Ferrell, 1996; but see Juslin, Winman, & Olsson, 2000; Merkle, 2009). The pervasiveness of this effect suggests that the hard-easy effect is a robust phenomenon of confidence processing and thus difficult to overcome. A corresponding phenomenon is observed across individuals, where overconfidence is found to increase with incompetence across several domains (the “unskilled-and-unaware-of-it” phenomenon; Kruger & Dunning, 1999). Though such changes in metacognitive readout are now well documented, their origin in the decision-making process remains poorly understood.

The goal of this study was to provide insight into the role of evidence reliability as a cue to decision confidence, both in terms of metacognitive sensitivity and metacognitive bias. To this end, we made use of a task developed by de Gardelle and Summerfield (2011), in which participants judged the average color of eight different-hued patches as being on average more red or more blue. This task requires the integration of multiple sources of evidence (i.e., color patches) toward a categorical decision (red vs. blue). The difficulty of this decision depends in part upon the strength of the evidence—that is, whether the average color of the patches falls near to or far from the category boundary. However, difficulty can also be manipulated orthogonally by changing the reliability of evidence, in terms of its variance across colored patches—the eight patches can be either relatively dissimilar or homogeneous in hue (high vs. low variance)—which de Gardelle and Summerfield (2011) showed affects decision speed and accuracy independent of evidence strength, with higher variance making decisions more difficult. Of critical interest was how these orthogonal manipulations of decision difficulty—evidence strength and evidence reliability—would affect decision confidence. We hypothesized that evidence reliability (manipulated via changes in variance) more than evidence strength would serve as a crucial cue to inform confidence. We tested this hypothesis in two ways: First, we predicted that evidence variance would have a larger impact on confidence than evidence strength when compared across two conditions that were matched for task difficulty but which differed in the source of that difficulty—weak evidence versus high variance evidence. Second, through regression analysis, we tested the prediction that evidence variance would explain changes in confidence over and above its effect on task performance. We then used signal detection theory (SDT) approaches to assess formally the impact of evidence strength and evidence reliability on metacognitive sensitivity and bias. To foreshadow, our findings suggest that evidence variance acts as a strong and useful cue to participants, affecting metacognitive readout so that their confidence judgments more accurately map their subjectively experienced certainty to their objective accuracy.

## Method

### Participants

After replacing 1 participant due to apparently random use of the confidence scale, the final sample comprised 20 participants, 14 of whom were female, with ages ranging from 18 to 25 years. All participants had normal or corrected-to-normal vision and—according to self-report—intact color vision. The experiment lasted approximately 90 min. Participants received course credit (*N* = 6) or money (£12; *N* = 15) as compensation. All testing was approved by the local research ethics committee.

### Task and Procedure

On each trial, participants performed a perceptual judgment and then shortly afterward indicated their degree of confidence that their perceptual judgment was correct. The perceptual task was to judge the average color of eight shapes presented simultaneously on a computer screen, determining whether this color was on average more red or more blue. The shapes were spaced regularly around a fixation point (radius ∼2.8° visual arc), and also varied along an irrelevant dimension (form, varying from square to circle) that did not affect the results and is not discussed further (cf. de Gardelle & Summerfield, 2011). The color judgment task can be made difficult in two distinct ways: first, by reducing the mean of the distribution (i.e., using colors that are, on average, purple hues rather than clearly red or blue) and, second, by increasing variance in the distribution of colors (i.e., using colors that are a mix of red, blue and purple rather than a homogeneous hue). The latter factor is our experimental manipulation of evidence variance. Of interest was the impact of manipulation of evidence mean and variance on decision confidence.

**Figure 1 fig1:**
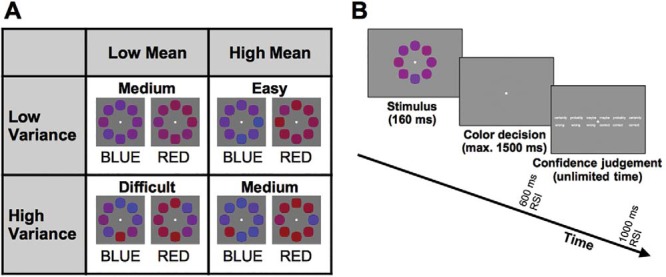
(A) Sample stimuli, showing the four difficulty conditions in the 2 (mean) × 2 (variance) design. Color values are made more extreme for illustrative purposes. (B) Design of the color task; participants had to indicate whether an array of eight colored shapes was on average more red or more blue by pressing the left or right response key. After making their response, the confidence scale was presented on screen and participants were given unlimited time to choose how confident they were that their last response was correct. RSI: response-stimulus interval; max. = maximum.

Factorial crossing of the two experimental factors results in four conditions of varying difficulty (Figure 1A), which were presented randomly interleaved within blocks. Stimulus mean and stimulus variance parameters of our experimental stimuli were carefully controlled such that the sample presented on any given trial very closely matched the mean and variance of the theoretical distributions from which they were drawn. The task is easy when stimulus mean is high (on average the color is very red or very blue) and stimulus variance is low (all stimulus elements exhibit this difference). Conversely, the task is very challenging when stimulus mean is low (the average color is “purplish red” or “purplish blue”) and variance is high. The other two conditions were of intermediate difficulty, but due to different stimulus characteristics: *Low mean, low variance* is difficult because the color information, though relatively homogeneous, does not clearly favor one option over the other. *High mean, high variance* is difficult because the evidence, though on average falling far from the category boundary, is noisy and unreliable. These two medium difficulty conditions were matched in terms of difficulty using a staircase procedure (see the online supplemental material). The comparison between these conditions was of critical interest, as it could reveal the impact of evidence mean and variance on confidence while controlling for primary task difficulty.[Fig-anchor fig1]

A typical sequence of trial events is shown in Figure 1B: Participants were shown the stimulus for 160 ms. They then made a speeded response to indicate their judgment of whether the average color of the stimulus was red or blue, with a time limit of 1,500 ms. Trials exceeding this time were counted as misses and a warning message instructed them to respond faster. After a 600-ms response-stimulus interval, a confidence scale was presented and participants indicated how confident they were regarding the correctness of their response by pressing one out of six keys. The confidence scale ranged from “certainly correct” to “certainly wrong.” Participants were given unlimited time for their judgments. The stimulus for the next trial appeared 1,000 ms after the confidence judgment.

Participants completed extensive training in the task, both with and without confidence judgments (512 to 704 trials), during which an adaptive procedure was used to match the medium conditions with regard to reaction times (RTs) and error rates (combined in the form of an efficiency measure: median correct RT divided by accuracy; see the online supplemental material for further details regarding the successful matching of difficulty of the two medium conditions). Participants then completed 16 experimental blocks of 64 trials each in which they performed the perceptual task followed by a confidence judgment. Prior to each block, participants completed 16 color-judgment trials without confidence ratings and instead with auditory feedback to help them maintain a stable color-discrimination criterion throughout the experiment. During the main part of the blocks, feedback was not given. Median correct RTs and error rates for the two color categories were shown on screen after the completion of each block.

Stimuli were presented on a 20-inch CRT monitor with a 75-Hz refresh rate using the MATLAB Toolbox Psychtoolbox3 with a 70-cm viewing distance. All responses were made with a USB keyboard. The color judgments were made with the *c* or *n* key (left or right thumb). Confidence responses were made with the upper number row (keys 1, 2, 3, 8, 9, and 0) using the index, middle, and ring fingers of the two hands. The direction of the confidence scale and mapping of colors to response keys were counterbalanced across participants.

### Data Analyses

The first set of analyses aimed to replicate previously observed effects of stimulus mean and variance on perceptual judgments and, crucially, to confirm that the two intermediate conditions were successfully matched for difficulty. This was assessed in terms of both RTs, error rates, efficiency, as well as using drift-diffusion model (DDM) fits that combine RT and error data to reveal latent variables (drift rate, *v*, and boundary separation, *a*) indicative of key features of the underlying decision process. To assess matching of the conditions in question, null effects were also analyzed using Bayesian statistical methods that permit estimating the probability with which the null hypothesis is true given the data (Morey & Rouder, 2014; Rouder, Speckman, Sun, Morey, & Iverson, 2009). For relevant analyses of variance and *t* tests, Bayes factors (*BF*s) are thus reported with interpretative guidelines proposed by Kass and Raftery (1995). According to these guidelines, values below the cutoff value between 1 and 3 fall into the category of “not worth more than a bare mention,” whereas values between 3 and 20 are considered to be “positive” evidence in favor of the hypothesis in question. Values between 20 and 150 are “strong” evidence, and above 150 are classified as “very strong” (Kass & Raftery, 1995, p. 777).

The key analyses focused on confidence judgments made shortly after each perceptual decision. For these analyses, confidence ratings were treated as an interval scale, as is typical in previous research (e.g., Baranski & Petrusic, 1998), by coding the six verbal categories from 1 (*certainly wrong*) to 6 (*certainly correct*). The objective of the first analysis of confidence judgments was to assess the impact of evidence mean and variance on average confidence ratings. A first test of our prediction that evidence variance would have a more pronounced effect on confidence than evidence mean was carried out by contrasting confidence across the difficulty-matched conditions with *low mean, low variance* and *high mean, high variance*. To look ahead briefly, the predicted reduction in confidence in the *high mean, high variance* condition was indeed apparent in the data. A subsequent analysis was therefore conducted to explore further the nature of this difference. More specifically, we aimed to identify more formally the unique contributions of these experimental manipulations on confidence, independent of their effect on overall task performance (response speed and accuracy).

We thus used regression techniques to assess whether evidence mean and variance affected confidence independent of their influence on primary task performance, as a direct test of whether either aspect served as a cue to confidence. Regression models were fitted to each participant’s data, that is to the means of the conditions (eight data points: 2 mean × 2 variance × 2 colors), predicting confidence from RTs, objective accuracy, as well as stimulus mean and variance, and data fits of these differing models were contrasted.

We hypothesized that evidence reliability (variance) as a cue to decision confidence would primarily affect people’s metacognitive readout, that is the process of internally mapping from subjectively experienced certainty to expressed confidence. A change in this mapping would correspond to a shift in metacognitive bias. We therefore applied well-established SDT techniques to disentangle the impact of evidence mean and variance on the sensitivity and bias of confidence judgments. To this end, we evaluated the impact of evidence mean and variance on signal detection measures of metacognitive sensitivity—that is, participants’ ability to detect reliably their errors versus correct responses—and metacognitive bias—that is, participants’ overall tendency to be underconfident, well calibrated, or overconfident. Note that because we used a confidence scale with verbal labels, all cases of over- and underconfidence have to be interpreted as relative changes, that is participants’ overall shifts toward one or the other end of the confidence scale. We used the approach of Kornbrot (2006), based on Receiver Operating Characteristic (ROC) curves to calculate distribution-free measures of metacognitive sensitivity, *A*_ROC_, and metacognitive bias, *B*_ROC_, thereby circumventing problems that arise from the fact that the distributional assumptions necessary for a robust fit of SDT parameters are often violated for metacognitive responses (Barrett, Dienes, & Seth, 2013; Evans & Azzopardi, 2007; see also Fleming & Lau, 2014, for a review of different measures).

## Results

### Basic Perceptual Performance

Stimulus mean had a significant effect on both correct RTs, *F*(1, 19) = 79.31, *p* < .001, η_*p*_^2^ = .81, and error rates, *F*(1, 19) = 203.69, *p* < .001, η_*p*_^2^ = .91, with a higher mean leading to faster RTs and lower error rates. Stimulus variance also had a reliable effect on correct RTs, *F*(1, 19) = 72.52, *p* < .001, η_*p*_^2^ = .79, and error rates, *F*(1, 19) = 92.77, *p* < .001, η_*p*_^2^ = .83, with lower variance leading to faster RTs and lower error rates. This replicates the findings by de Gardelle and Summerfield (2011). There was no interaction between the two factors for correct RTs (*F* < 1), but there was an interaction effect for error rates, *F*(1, 19) = 11.94, *p* = .003, η_*p*_^2^ = .39. These effects are presented in Table 1.[Table-anchor tbl1]

### Matching of Medium Difficulty Conditions

Comparison of the two medium conditions indicated that they were well matched for difficulty but exhibited a small but consistent speed-accuracy trade-off. Thus, the conditions were matched in terms of the efficiency measure (*high mean, high variance*: 735 ms, vs. *low mean, low variance*: 722 ms; *t* < 1; *BF*_NULL_ = 3.18) and drift rate from a DDM that integrated RT and error rate to estimate difficulty (*high mean, high variance*: *v* = −.32, vs. *low mean, low variance*: *v* = −.33; *t* < 1; *BF*_NULL_ = 2.88). However, participants were reliably slower, *t*(19) = 3.89, *p* < .001; *BF* = 36.73, and more accurate, *t*(19) = 2.23, *p* = .038; *BF* = 1.74, in the *high mean, high variance* condition (mean RT = 680 ms, mean error rate = 11.9%) as compared to the *low mean, low variance* condition (mean RT = 649 ms, mean error rate = 14.9%). Correspondingly, DDM models revealed a reliable difference in boundary separation, with a more cautious threshold in the *high mean, high variance* condition (*a* = .15) as compared with the *low mean, low variance* condition (*a* = .14), a difference that was numerically small but very consistently observed across participants, *t*(19) = 3.9, *p* < .001, *BF* = 37.12, *d* = .87. Condition averages of efficiency, drift rate, and boundary separation are given in Table 1. Taken together, these effects suggest that our manipulations of evidence strength (mean color) and evidence variance (color variance) had their expected effect on perceptual judgments, and were matched in terms of the overall magnitude of their effects on task difficulty. However, the two medium difficulty conditions were not perfectly matched in every respect, exhibiting a small but consistent difference in speed-accuracy trade-off.

### Subjective Confidence and Objective Accuracy

**Figure 2 fig2:**
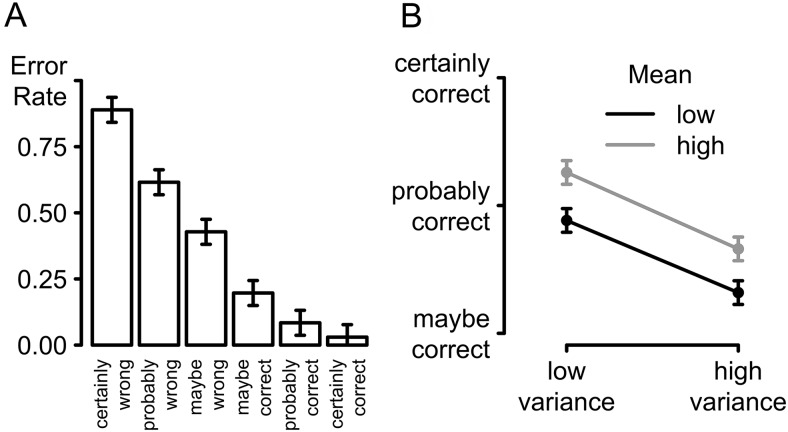
(A) Distributions of confidence levels displayed for correct and error trials separately. (B) Confidence for the four difficulty color conditions (together for both error and correct trials). All error bars are within-subject confidence intervals (Loftus & Masson, 1994).

Figure 2A plots the overall relationship between confidence and accuracy, pooling data across conditions, to show the expected monotonic decrease in error rates with level of confidence, with the highest error rates for trials reported as *certainly wrong* (*M* = 88.9%), and the lowest error rate for the trials reported as *certainly correct* (*M* = 3.0%). Across participants, confidence varied with accuracy, as expressed in Spearman rank correlations, which were found to be significantly different from zero (*r*s ≤ −.94, *p*s ≤ .005), except for one participant (*r* = −.21, *p* > .250), who did not have enough trials in the two lowest categories of the confidence scale to obtain a stable correlation estimate.[Fig-anchor fig2]

### Effects of Evidence Mean and Variance on Confidence

The mean level of confidence for each of the four conditions, aggregated over correct and error trials, is presented in Figure 2B (see the online supplemental material for a plot showing condition-averaged confidence for each participant separately). Both stimulus mean and stimulus variance reliably affected confidence: The greater the distance of the mean color of a stimulus array from the category boundary, the higher the confidence reported by participants, *F*(1, 19) = 92.89, *p* < .001, η_*p*_^2^ = .83. The higher the variance of a stimulus, however, the lower the confidence rating that followed the response to this stimulus, *F*(1, 19) = 71.45, *p* < .001, η_*p*_^2^ = .79. These two factors did not interact (*F* < 1).

Analysis focusing on the two medium conditions that were matched for overall difficulty confirmed our first key prediction: Participants were on average less confident in the *high mean, high variance* condition than in the *low mean, low variance* condition. This difference was observed for both correct, *t*(19) = 3.98, *p* < .001, *BF* = 44.24, and error trials, *t*(19) = 2.98, *p* = .008, *BF* = 6.36.

Several features of the results indicate that this difference in confidence between difficulty-matched conditions is not a consequence of the small speed-accuracy trade-off apparent in basic task performance (with participants responding slightly more cautiously in the *high mean, high variance* condition). First, and most obviously, the difference in confidence between the conditions is the opposite of what one would expect normatively given the difference in accuracy. That is, objective accuracy was higher in the *high mean, high variance* condition, which implies that participants should have been, if anything, more confident here rather than less. Correspondingly, formal models that explain confidence as a reflection of the evidence accumulation process predict that confidence should increase with response caution, tracking the increase in objective accuracy (Moreno-Bote, 2010; Vickers & Packer, 1982). Our results stand in contrast to this prediction.

Nor did the observed difference in confidence stem from subtle differences in RT across conditions, as might be predicted from theories proposing that confidence scales inversely with RT (Audley, 1960; Kiani et al., 2014; Zylberberg et al., 2012; see also Wilimzig, Tsuchiya, Fahle, Einhäuser, & Koch, 2008): The reduction in confidence for the *high mean, high variance* condition was also seen on error trials, which did not differ in RT across conditions (*t* < 1, *BF*_NULL_ = 3.71).

To further verify that the difference in confidence was not due to a difference in RTs between these two conditions, we looked specifically at participants for whom this difference in RTs was minimal. A median split on the difference in RTs isolated a subgroup of 10 participants, who exhibited no difference in correct RTs between the two medium difficulty conditions, *t*(9) = 1.09, *p* > .250, *BF*_NULL_ = 2.00. For these participants, we found greater accuracy in the *high mean, high variance* condition (10.7% vs. 15.4%), *t*(9) = 2.90, *p* = .018, *BF* = 3.96, but lower confidence in this condition. This lower confidence for the *high mean, high variance* relative to the *low mean, low variance* condition, was found both for correct trials, *t*(9) = 2.49, *p* = .034, *BF* = 2.35, and for error trials, *t*(9) = 2.78, *p* = .021, *BF* = 3.39. (Full factorial analysis of both median-split groups is presented in the online supplemental material. The results from these analyses further support the findings reported here.)

Altogether, therefore, we find a robust reduction in confidence in the *high mean, high variance* condition relative to the *low mean, low variance* condition, despite these conditions being well matched for overall difficulty. The observed difference in confidence appears to reflect intrinsic differences in the effects of stimulus mean and variance on the decision process, rather than being caused by the small speed-accuracy trade-off difference between the two conditions.

### Unique Contributions of Mean and Variance to Confidence

The preceding analyses suggest that high-variance evidence leads to lower confidence, and that this effect is present even when the high-variance condition is compared to a condition with low-evidence strength (low mean) that is matched in overall task difficulty.

**Figure 3 fig3:**
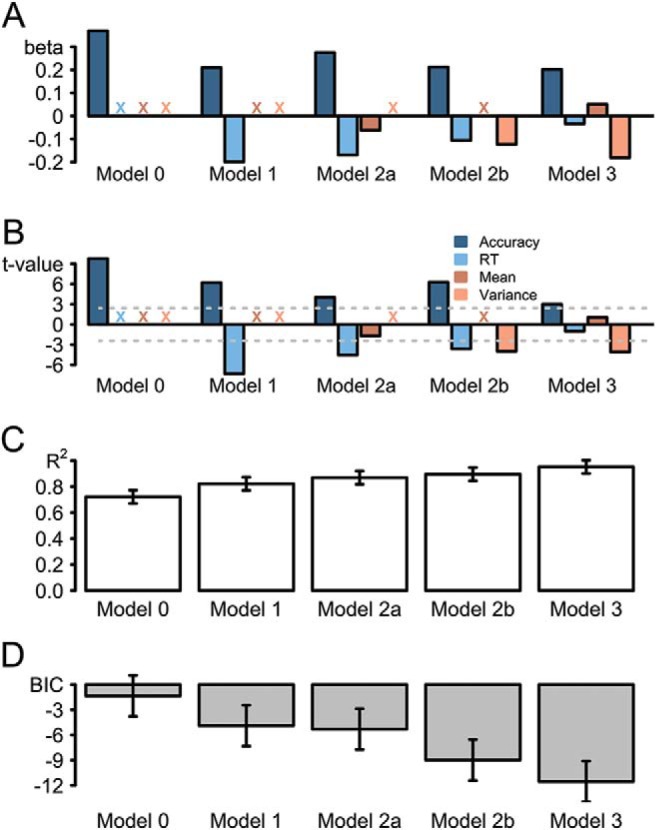
(A) Standardized regression coefficients for three different models to predict confidence. Colored *x*s reflect the respective parameters that were not considered in the regression model. (B) Signed *t* values for three different models to predict confidence. The horizontal dashed lines highlight the critical *t* values, so that model parameters above or below the dashed, horizontal lines are significantly different from zero. (C) Mean *R*^2^ and (D) mean *BIC*s for these models. RT = reaction time; *BIC* = Bayesian information criterion.

To shed further light on the nature of this effect, we used a more formal modeling approach to assess the unique contribution of evidence variance (as well as evidence strength) as a cue to confidence. To this end, we contrasted regression models that were fitted separately to each participants’ confidence judgment data, with models including first-order performance predictors (RT and accuracy) in addition to predictors reflecting changes in evidence mean and variance across conditions. For each participant, a regression model was fitted to eight data points, that is the four difficulty conditions crossed with the two color conditions. Here, we report second-order statistics based on the resulting standardized beta weights (Figure 3A).[Fig-anchor fig3]

A first pair of models indicated that accuracy (log-odds error rates; Zhang & Maloney, 2012) was a strong predictor of confidence—as one would expect given that confidence is expressed as a subjective estimate of accuracy—and that between-conditions differences in RT accounted for further variance in confidence (Models 0 and 1, respectively). Figure 3 presents the results from this model-comparison approach: Figure 3B presents the signed *t* values. The positive *t* value for the accuracy factor in Model 0 indicates that the more accurate a participant was, the more confident he or she was. Figure 3C shows the explained variance of the models, as expressed in *R*^2^. To allow for direct model comparisons, Figure 3D presents Bayesian information criteria (*BIC*s) for each model. The lower the *BIC* score, the better the model fit the data, as was the case for the more complex Model 1, *BIC*_M1_ = −4.89, if compared to Model 0, *BIC*_M0_ = −1.35. This difference in *BIC*s was reliable, *t*(19) = 2.98, *p* = .008.^1^ Thus, these basic models establish that, as one would expect given the findings reported above, confidence scaled with first-order performance.

Of critical interest were the unique contributions of stimulus mean and variance, that is, whether these factors accounted for changes in confidence above and beyond the first-order performance predictors. A separate model each was thus fitted to also include these factors. Model 2a added stimulus mean as a predictor of confidence. In this model, the regression weight for the factor of stimulus mean was not significantly different from zero, *t*(19) = 1.70, *p* = .106, and correspondingly the reduction in *BIC* (to −5.31) for the model as a whole was not significant when compared to Model 1 (*t* < 1). In contrast, stimulus variance was a reliable predictor of confidence, *t*(19) = 3.98, *p* < .001, and including this predictor improved model fit compared to Model 1, *BIC*_M2b_ = −8.98, *t*(19) = 3.16, *p* = .005, indicating that stimulus variance explains between-conditions differences in confidence over and above first-order performance predictors, consistent with the key hypothesis of this study. Finally, the best-fitting model included all four predictors, Model 3, *BIC*_M3_ = −11.54. The *BIC* scores of this full model were reliably lower than Model 2a, *t*(19) = 4.56, *p* < .001. This finding is consistent with the idea that evidence variance but not evidence mean has a reliable effect on confidence above and beyond its effect on basic task performance. Therefore, including evidence variance in the model as a predictor improves the overall fit of the model. The *BIC* scores of Model 3 were marginally significantly different from Model 2b, *t*(19) = 2.08, *p* = .051, but inspection of Figure 3A indicates that this marginal effect does not indicate a significant effect of evidence mean on confidence, but rather that the model fit is improved by a rebalancing of the effects of accuracy, RT, and condition (high/low mean) on confidence when the three factors are considered simultaneously.

In a final set of regression analyses, we confirmed that these patterns of results were preserved when accuracy and RT were not included as predictors in the regression model, with greater impact on confidence of variance over mean. For this analysis, the regressors were again standardized so that the influences on confidence can be directly compared. Both the regression weights for mean, β_mean_ = 0.19, *t*(19) = 9.65, *p* < .001, and variance, β_variance_ = −0.31, *t*(19) = 8.44, *p* < .001, are significantly different from zero. Comparing their unsigned values, we found that the absolute weight of variance was numerically greater than the absolute weight of mean on confidence, in reliable manner across participants, *t*(19) = 3.47, *p* = .003.

Taken together, these analyses suggest that whereas evidence mean and evidence variance both affect confidence indirectly via their effect on task difficulty (and thus accuracy and RT), only evidence variance had an effect on subjectively rated confidence over and above these first-order performance effects. These findings support the view that evidence reliability serves as a crucial cue to inform decision confidence.

### Confidence Sensitivity and Confidence Bias: SDT Model Fits

**Figure 4 fig4:**
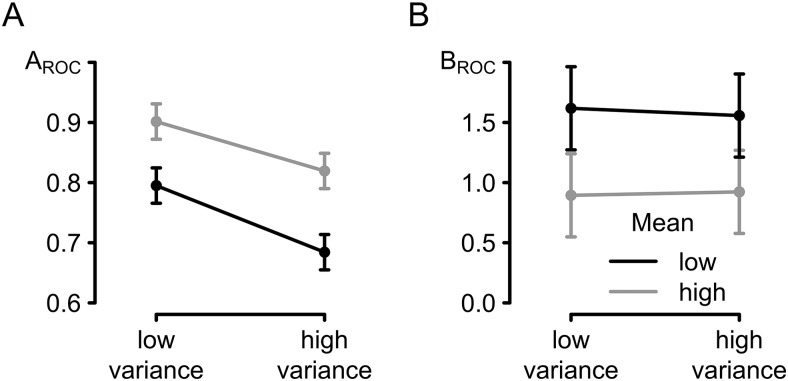
Nonparametric Type II SDT parameter estimates as a function of difficulty. (A) *A*_ROC_ expresses metacognitive sensitivity. (B) *B*_ROC_ expresses metacognitive bias. All error bars are within-subject confidence intervals (Loftus & Masson, 1994). SDT = signal detection theory; ROC = receiver operating characteristic.

The preceding analyses demonstrate that evidence variance has a larger effect on decision confidence than evidence mean. Two detailed features of the results suggest that the differential effect of mean and variance primarily reflect differences in metacognitive bias rather than sensitivity—that is, through up- and downregulation of confidence ratings (metacognitive readout) rather than reduced ability to discriminate correct and incorrect perceptual judgments. First, confidence was reduced for both correct and error trials in the *high mean, high variance* condition, whereas a reduction in sensitivity would imply an increase in confidence following errors in this condition. Second, the preceding regression analyses indicate that evidence variance reduces confidence below the level that would be expected from objective performance measures. To test this conclusion more directly, our final analysis applied SDT approaches to disentangle the impact of experimental manipulations on metacognitive sensitivity (participants’ ability to discriminate between their correct and incorrect responses) and metacognitive bias (participant’s overall tendency to express high or low confidence in their decisions). Figure 4 presents SDT measures for the four difficulty conditions. Figure 4A presents metacognitive sensitivity, *A*_ROC_, that is how accurate participants were in distinguishing between correct and error trials. Across all four conditions and all participants, *A*_ROC_ values were different from chance performance of 0.5 (minimum = .55; maximum = 1.00). Four paired *t* tests with corrected alpha levels revealed that these values were indeed reliably different from 0.5 (*t*s ≥ 11.8, *p*s < .001). Both stimulus mean and variance had a significant influence on sensitivity: The closer the mean color of the stimulus to the category boundary, the worse participants were at discriminating their own correct from their error responses, *F*(1, 19) = 53.05, *p* < .001, η_*p*_^2^ = .74, with a corresponding effect observed when perceptual task difficulty was increased via increasing variance in hue across stimulus elements, *F*(1, 19) = 41.46, *p* < .001, η_*p*_^2^ = .69. These two factors did not significantly interact, *F*(1, 19) = 1.40, *p* > .252, η_*p*_^2^ = .07. There was, however, no significant difference in measured sensitivity of confidence judgments between the two medium conditions, *t*(19) = 1.05, *p* > .308, *BF*_NULL_ = 2.66. Thus, as one would expect given the condition differences in first-order performance, both stimulus mean and variance influenced how well participants distinguished between their correct and error responses. Critically, there was no difference in metacognitive sensitivity between the two medium conditions for which performance had been carefully matched. Caution is advised when comparing conditions that are not matched for accuracy, though (Fleming & Lau, 2014). We therefore repeated the analysis using a measure of metacognitive accuracy that takes such differences into account (meta*-d*′; Maniscalco & Lau, 2012). This analysis is reported in the online supplemental material, mirroring the findings reported here.[Fig-anchor fig4]

The second analysis focused on metacognitive bias, that is the overall tendency to classify responses as correct or incorrect independent of objective accuracy. If participants rate their responses more likely to be correct than they are objectively, they are overconfident. If, however, they rate them as less likely to be correct than they are, then they are underconfident. Figure 4B presents the metacognitive biases for the four conditions. Only stimulus mean had a significant effect on metacognitive bias, *B*_ROC_: Participants tended to express relatively higher confidence—that is higher than adequate regarding changes in objective accuracy—when mean stimulus color fell close to the category boundary than when it fell far from the boundary, *F*(1, 19) = 26.68, *p* < .001, η_*p*_^2^ = .58. Surprisingly, however, stimulus variance showed no such effect on metacognitive bias (*F* < 1). The two factors also did not interact significantly (*F* < 1). The results from this analysis were further supported by the fact that there was most evidence in favor of a Bayesian model with just the main effect of stimulus mean, *BF* = 125.56 (tested against a null model). Critically, the two medium conditions, matched for accuracy, were found to be significantly different in terms of the metacognitive bias, *B*_ROC_, *t*(19) = 4.23, *p* < .001, *BF* = 73.52, with a significantly higher metacognitive bias for the *low mean, low variance* conditions compared to the *high mean, high variance* condition.

Taken together, these findings indicate that for stimulus mean (but not stimulus variance), people struggle to assess the difficulty of a task at hand, failing to shift their confidence ratings to match changes in their objective accuracy across conditions. As a consequence, participants were relatively overconfident when the task was more difficult. This finding is reminiscent of the hard-easy effect—the often-replicated observation of relative overconfidence when the task is difficult. In contrast, participants’ confidence ratings exhibited no corresponding hard-easy effect when difficulty was manipulated via change in stimulus variance: Participants appropriately shifted their confidence ratings to match changes in their objective accuracy across low- versus high-variance conditions. These analyses provide further support for the hypothesis that variability in stimulus evidence provides a crucial cue to confidence, here allowing participants to overcome the otherwise-pervasive hard-easy effect to the difficulty of the task if this difficulty is caused by changes in stimulus variance.

## Discussion

In the present study, we manipulated evidence strength and reliability in a perceptual decision-making task to address two key questions. The first question was whether evidence strength and reliability would have differential effects on decision confidence. We found that participants were less confident on trials with unreliable evidence (high stimulus variance) than on trials with weak evidence (low stimulus mean), despite these conditions being matched in terms of task difficulty, consistent with our key prediction that evidence variance—a signal of evidence reliability—would have a more pronounced effect on confidence than evidence strength. This hypothesis was furthermore supported by regression analyses which indicated that the unique contribution of evidence variance on confidence existed over and above the effect this factor had on first-order task performance.

These findings have strong theoretical implications for how decision confidence is formed, and highlight the role of evidence reliability as a crucial cue to confidence. More specifically, our results suggest that decision confidence is not a direct readout of the evidence leading to the initial decision, as would be predicted by dominant theories in the decision-making literature. Instead, confidence appears to be sensitive to multiple other cues such as the perceived reliability of the evidence on which a decision is based. In this regard, our conclusions align with current theories in other domains, in particular research on metacognition in memory (*metamemory*), where multicue models provide the prevailing account of metacognitive judgments (see Koriat & Levy-Sadot, 2001; Nelson, Gerler, & Narens, 1984, for a similar suggestion regarding feeling-of-knowing judgments).

Our second key question was whether evidence reliability is used specifically as a cue to inform a second crucial feature of confidence: the degree to which confidence judgments are calibrated to reflect objective performance versus exhibit systematic bias toward under- or overconfidence. To date, this aspect of confidence has received less attention in formal theories of decision making, with the primary focus of past research being on metacognitive sensitivity. To provide insight into this aspect of confidence, we assessed whether our two difficulty manipulations would have differing effects on metacognitive bias. We found that participants exhibited relative overconfidence when evidence was weak (i.e., mean stimulus color fell close to the category boundary) than when it was strong (i.e., mean stimulus color fell far from the boundary)—reminiscent of the hard-easy effect that has been consistently reported in prior research (Baranski & Petrusic, 1994; Drugowitsch et al., 2014; Gigerenzer et al., 1991; Merkle & Van Zandt, 2006). In contrast, no hard-easy effect was apparent when comparing conditions differing in evidence reliability: Metacognitive bias did not increase in trials with high stimulus variance relative to trials with low variance. Thus, participants’ ratings of subjective confidence adjusted appropriately to changes in objective task performance only as a function of evidence reliability, not evidence strength.

Why should evidence reliability influence confidence more than evidence strength? One intriguing hypothesis is that taking into account the reliability of evidence is a straightforward process in the sense that differences in stimulus variance reflect the “native language” of confidence. According to this view, which was inspired by the hypothesis that uncertainty can be represented explicitly in terms of the variance in firing rate across neural populations (Beck et al., 2008; Ma et al., 2006), participants can very easily read out signal reliability and transform it into decision confidence, correctly accounting for changes in difficulty due to evidence reliability (Yeung & Summerfield, 2014). Indeed, there have been findings supporting the notion that mental representations are stored and accessed as a probability distribution of activations rather than a point estimate (Bach & Dolan, 2012; Beck et al., 2008; Fiser, Berkes, Orbán, & Lengyel, 2010; Ma et al., 2006) and it has been proposed that confidence could be a readout of such reliability estimates (Meyniel et al., 2015; Pouget et al., 2016; Yeung & Summerfield, 2014). On the contrary, it may be that participants underestimate the detrimental effect of stimulus mean on accuracy because factoring changes in evidence strength into subjective confidence requires an additional transformation compared to taking into account reliability. In this regard, a key point is that assessing whether a stimulus has a low- or high-stimulus mean requires an additional step of comparing the mean color of the stimulus to an internal decision boundary—that is, evidence strength in our task is a relative rather than absolute factor. Moreover, this decision boundary could presumably be noisily represented. Using evidence strength as a cue to confidence may therefore be computationally more complex, and correspondingly less reliable, compared to evidence reliability. Note that this finding stands in contrast to the results of a recent study by Kvam and Pleskac (2016; see also Brenner, Griffin, & Koehler, 2012; Griffin & Tversky, 1992): Kvam and Pleskac found that evidence strength had a three-times higher influence on confidence compared to evidence weight. However, it should be noted that in the case of their study, evidence weight was not operationalized as the variability of the available evidence on which the decision is based, but rather the overall amount of evidence. The findings are therefore not directly comparable to ours.

Taken together, the approach chosen in the present study allowed us to disentangle the unique effects different cues have on metacognitive sensitivity and bias, in particular highlighting the importance of studying such biases and the metacognitive readout processes that give rise to them. This importance is furthermore highlighted by the pervasiveness of hard-easy effects and the unskilled-and-unaware-of-it phenomenon. Moreover, metacognitive biases have been suggested to underpin effective communication in group decision making: A recent study by Fusaroli and colleagues (2012) showed that participants performed better in a collective decision-making task if the verbal descriptions of their confidence states matched, that is if they used the same linguistic expressions. Given these findings, it could be argued that similar benefits could be caused by a matching in the metacognitive readout: Participants who assign the same confidence label to the same internal level of certainty should be able to benefit much more from communicating their confidence levels, which require no further transformation and can instead be interpreted straightforwardly to discount contributions from communication partners with little or no confidence (Bang et al., 2014). Whether this is indeed the case remains to be addressed in future studies.

Our findings add to a growing corpus of data on the effects of evidence reliability on decision confidence (de Gardelle & Mamassian, 2015; Irwin et al., 1956; Spence et al., 2016; Zylberberg et al., 2014). In an early study, Irwin and colleagues (1956) found that participants’ confidence in a number averaging task is affected by the variance of the numbers. Recently, Spence et al. (2016) showed using a dot-motion discrimination task that this effect of variance can be observed even across conditions that are matched in terms of first-order accuracy, following a partly similar logic to the present study. The results reported here replicate these findings, and extend them in several important ways: We directly compare the effect of evidence reliability with those of evidence strength to investigate their unique contributions to confidence while accounting for their impact on first-order task performance; we show that differences between strength and reliability effects on confidence persist even when conditions are carefully matched for performance across both RT and accuracy measures; and we consider confidence in both correct and error responses, and apply signal detection theoretic approaches, that allow us to disentangle the influence of evidence strength and reliability on metacognitive bias as well as sensitivity. Altogether our results identify a clear effect of evidence reliability on decision confidence, but critically one that we characterize as reflecting an adaptive scaling of metacognitive readout so that confidence shifts appropriately with changes in objective accuracy across conditions, scaling that is not achieved when task difficulty is manipulated via change in evidence strength where the typical hard-easy effect is observed.

While aligning with some previous reports (Irwin et al., 1956; Spence et al., 2016), our findings apparently diverge from those of a recent study (Zylberberg et al., 2014) which found that larger stimulus variance can lead to higher levels of confidence, rather than lower as observed here. This differing effect of variance on confidence might reflect specific features of their experimental design, such as the complexity of the display (with many more elements than the eight used here) or the nature of the judgment required (about line orientation rather than color). However, we suspect that the critical feature of Zylberberg et al. (2014)’s study was their focus on trials in which evidence strength was zero or near zero (i.e., the mean orientation of lines in the display fell exactly on, or very close to, the category boundary of the perceptual judgment task). Sanders, Hangya, and Kepecs (2016) have shown that in this special case, overconfidence is normatively justified if the observer does not know a priori that the task is impossible, because any evidence used to make the decision should increase confidence (even if, unbeknownst to the observer, this evidence reflects noise rather than true signal). To the extent that participants mistake noise as useful signal, as proposed by Zylberberg et al. (2014), increased variance should therefore paradoxically increase confidence when the task is objectively impossible because it will increase the likelihood of sampling evidence that lies far from the category boundary. In contrast, when the mean of the stimulus display more clearly favored one of the choice options in Zylberberg et al. (2014)’s task, the effect of stimulus variance reversed and participants were less confident when the variability in a line orientation stimulus was higher, as expected from their performance (and consistent with our results). This discussion brings to the fore the question of how stimulus reliability is estimated, and why systematic mis-estimation may sometimes occur (as in Zylberberg et al.’s study), a question that in future research will usefully be informed by emerging ideas regarding the neural representation of uncertainty and its relation to confidence (Pouget et al., 2016).

In conclusion, the present study supports the hypothesis that decision confidence is affected by a range of different cues that includes the perceived reliability of the evidence used to make the initial decision. Our findings indicate that less reliable evidence leads to lower confidence, and does so to a greater extent than changes in evidence strength that have equivalent effects on first-order performance. As a consequence, only changes in evidence reliability led to an appropriate downscaling of confidence when the task became more difficult, and we propose this is due to the fact that evidence reliability is the “native language” of confidence. Here, we attribute the effects of evidence reliability to changes in metacognitive bias, that is participants’ mapping from subjectively experienced certainty to expressed confidence. The finding of such changes in metacognitive biases point to the importance of studying the metacognitive readout process. This readout process has often been overlooked in studies of decision confidence, which have more commonly focused on metacognitive sensitivity. Future studies should focus on how different cues—relying both on privileged access to the decision itself, but also on simple heuristics—affect metacognitive sensitivity and bias and whether ways could be found to manipulate participants to selectively base their confidence judgments on some cues but not others, in a context-dependent fashion. Arguably, some cues are more valid than others (Gigerenzer et al., 1991), depending for instance on contextual factors such as whether speed or accuracy are more important in a task. It would thus be worthwhile to develop a method to train participants to tune their attention to the currently most valid cues for confidence. Such an approach would ultimately result in people becoming more metacognitively accurate, which could in turn lead to improved cognitive control (Logan & Crump, 2010; Yeung & Summerfield, 2014).

## Supplementary Material

10.1037/xhp0000404.supp

## Figures and Tables

**Table 1 tbl1:** Condition Means (and Standard Errors) for RTs, Error Rates, Efficiency Scores, Drift Rates, and Boundary Separation Scores

Condition	Stimulus mean	Stimulus variance	RT (ms)	Error rate (%)	Efficiency (ms)	Drift rate (*v*)	Boundary separation (*a*)
Easy	High	Low	599 (21)	5.3 (.9)	601 (21)	−.52 (.04)	.61 (.16)
Medium	High	High	680 (28)	11.9 (1.3)	735 (35)	−.32 (.02)	.15 (.01)
Medium	Low	Low	649 (25)	14.9 (1.3)	722 (32)	−.33 (.02)	.14 (.01)
Difficult	Low	High	729 (31)	24.9 (1.1)	926 (49)	−.17 (.01)	.15 (.01)
*Note*. RT = reaction time.							

## Footnotes

1 The reader is referred to a recent study by Stephan, Penny, Daunizeau, Moran, and Friston (2009) on Bayesian model selection for group studies for more details on the approach of comparing *BIC*s using *t* tests.
