# Supplementary material for: The Impact of Evidence Reliability on Sensitivity and Bias in Decision Confidence
Source: J Exp Psychol Hum Percept Perform. 2017 Apr 6;43(8):1520–31. doi: 10.1037/xhp0000404 (PMC5524444; doi:10.1037/xhp0000404)
Supplement: Supplementary file 1 [file zfn999173601so1.doc]

**Supplemental Materials**

**The Impact of Evidence Reliability on Sensitivity and Bias in Decision Confidence**

**by A. Boldt et al., 2017, *JEP: Human Perception and Performance***

**http://dx.doi.org/10.1037/xhp0000404**

**Staircase**

The level of stimulus mean in the *low mean, low variance* condition was varied so that performance in this condition was matched with performance in the *high mean, high variance* condition. Matching was done specifically with reference to an efficiency measure, median correct RT divided by accuracy (inverse efficiency score *IES*; Bruyer and Brysbaert, 2011). More precisely, at the beginning of the practice staircase blocks (blocks 2 to 8), the *low mean* condition was adjusted whenever the two medium conditions were not matched with regard to the efficiency measure. Matching was assessed across the preceding block. If there was a difference of at least 10 ms/acc, the *low mean* condition would be increased or decreased by 2% of the initial difference between *low* and *high mean*. If the difference was at least 50 ms/acc, the change would be 5%, and for 100 ms/acc or more, the change would be 10%. After these adjustment blocks, the experimenter decided whether additional adjustment blocks were needed, based on visual inspection of RTs and error rates for the two medium conditions, which were shown on screen. For example, if performance in the two conditions was converging but not yet quite matched, the experimenter would decide to include yet another practice block for this particular participant. Participants completed seven to ten adjustment blocks in total, during which a feedback tone was played every time they committed an error. They then completed one block of task practice in which the confidence scale was presented for the first time. After completion of this block, frequencies for each confidence category were displayed on screen and the experimenter discussed these values with the participants, encouraging them to use the full scale.

Both stimulus mean, *F*(1, 19) = 93.69, *p* < .001, η^2^*_p_* = .83, and variance, *F*(1, 19) = 74.24, *p* < .001, η^2^*_p_* = .80, showed a reliable main effect on efficiency. The two factors interacted reliably, *F*(1, 19) = 25.25, *p* < .001, η^2^*_p_* = .57. As stated in the main text, matching of the medium-difficulty conditions was successful (*high mean, high variance*: 735 ms versus *low mean, low variance*: 722 ms; *t* < 1; *BF_NULL_* = 3.18).

**Participant-wise confidence effects**

Figure S1 shows average confidence as a function of difficulty for each individual participant.

**Median-split Analysis**

**Basic perceptual performance for the two median-split groups.** For the median-split group with the small difference in correct RTs, we found a main effect of stimulus mean on correct RTs, *F*(1, 9) = 38.87, *p* < .001, η^2^*_p_* = .81, and error rates, *F*(1, 9) = 110.3, *p* < .001, η^2^*_p_* = .92. The main effect of stimulus variance was replicated for both correct RTs, *F*(1, 9) = 54.56, *p* < .001, η^2^*_p_* = .86, and error rates, *F*(1, 9) = 40.60, *p* < .001, η^2^*_p_* = .82. The two factors showed an interaction only for error rates, *F*(1, 9) = 6.46, *p* = .032, η^2^*_p_* = .42, and not for correct RTs, *F* < 1.

The other median-split group showed a reliable difference in correct RTs between the two medium conditions, *t*(9) = 5.75, *p* < .001, but no difference in error rates, *t* < 1, *BF_NULL_* = 2.77. This group also showed a main effect of stimulus mean on both correct RTs, *F*(1, 9) = 41.18, *p* < .001, η^2^*_p_* = .82, and error rates, *F*(1, 9) = 87.18, *p* < .001, η^2^*_p_* = .91, as well as a reliable effect of stimulus variance on correct RTs, *F*(1, 9) = 47.48, *p* < .001, η^2^*_p_* = .84, and error rates, *F*(1, 9) = 59.15, *p* < .001, η^2^*_p_* = .87. Once more, a reliable interaction was found only for error rates, *F*(1, 9) = 5.69, *p* = .041, η^2^*_p_* = .39, and not for correct RTs, *F* < 1. These data are furthermore presented in Table S1.

**Effects of evidence mean and variance on confidence for the two median-split groups.** For the sake of completeness, confidence analysis for the two median-split groups will be reported here. These data are also presented in Table S1. For the median-split group with the small difference for correct RTs, there was a reliable main effect of stimulus mean, *F*(1, 9) = 49.79, *p* < .001, η^2^*_p_* = .85. Participants were more confident in the *high mean* condition on correct trials, and less confident in the *high mean* condition on error trials. This pattern was also reflected in a reliable interaction between stimulus mean and accuracy, *F*(1, 9) = 74.86, *p* < .001, η^2^*_p_* = .89. There was also a reliable effect of accuracy, *F*(1, 9) = 83.37, *p* < .001, η^2^*_p_* = .90, with higher confidence on correct, *M* = 5.0, compared to error trials, *M* = 3.3. There was, however, no reliable effect of stimulus variance, *F* < 1. Stimulus mean and variance showed a reliable interaction, *F*(1, 9) = 7.98, *p* = .020, η^2^*_p_* = .47, as well as stimulus variance and accuracy, *F*(1, 9) = 18.38, *p* = .002, η^2^*_p_* = .67. There was also a marginally significant three-way interaction, *F*(1, 9) = 3.89, *p* = .080, η^2^*_p_* = .30.

For the median-split group with the larger difference in correct RTs, there was no reliable effect of stimulus mean, *F*(1, 9) = 2.69, *p* = .135, η^2^*_p_* = .23, but instead of stimulus variance, *F*(1, 9) = 6.44, *p* = .032, η^2^*_p_* = .42. This effect was again in opposite directions for correct and error trials, with higher confidence for *low variance* trials compared to *high variance* trials for corrects and the reverse pattern for error trials. This effect was also reflected in a significant interaction between stimulus variance and the objective accuracy of the trial, *F*(1, 9) = 15.08, *p* = .004, η^2^*_p_* = .63. This group also showed an effect of objective accuracy with higher confidence for correct, *M* = 4.9, compared to error trials, *M* = 3.4, *F*(1, 9) = 69.59, *p* < .001, η^2^*_p_* = .89. Stimulus mean and accuracy showed a reliable interaction, *F*(1, 9) = 33.14, *p* < .001, η^2^*_p_* = .79, in the same direction as for the other median-split group. No other interactions were significant, *Fs* < 1. For this median-split group, there was a reliable difference in confidence between medium conditions only for correct, *t*(9) = 3.22, *p* = .011, but not for error trials, *t*(9) = 1.38, *p* = .200, *BF_NULL_* = 1.53.

**Metacognitive Efficiency Analysis**

One of our key analyses asks whether metacognitive sensitivity—as reflected in estimates of *A_ROC_*—varies as a function of stimulus mean and variance. This was indeed the case, people were found to be more metacognitively accurate when the stimulus mean was high and when stimulus variance was low. These findings clearly follow the differences is first-order performance, and indeed several studies have noted that metacognitive sensitivity will always—at least to some extent—depend on first-order sensitivity, *d′* (for a review, see Fleming & Lau, 2014). This dependency does not complicate the interpretation of our key contrast of the two medium conditions of matched difficulty. However, to rule out the possibility that stimulus mean or variance had a residual effect on metacognitive accuracy above and beyond its effect on first-order performance, we repeated the analysis using an SDT estimation method that permits comparison of metacognitive judgments across conditions that differ in basic task performance. Maniscalco and Lau (2012) have proposed this method to estimate type-II SDT sensitivity relative to their underlying type-I parameters. Such corrected type-II sensitivity measures (*meta-d′*) can then be compared to type-I sensitivity (*d′*) by calculating the ratio of the two measures: Metacognitive efficiency (*M-ratio*) expresses how well participants differentiated between their own correct responses and errors while taking first-order performance into account. This parameter can be interpreted as follows: A value of 1.0 reflects that participants used all information from the primary decision to form their metacognitive judgment. A value of .7 means that they only used 70%. Values larger than 1.0 express that participants used more information for their second-order judgment than in their initial decision, for example because they continue to process information even after their initial choice and this continued processing helps inform their subsequent confidence judgement. *M-ratio* is therefore an index of the degree to which metacognitive sensitivity (*meta-d′*) falls below or exceeds what would be expected given primary task sensitivity (*d′*). Here, we report the common logarithm of *M-ratio* to correct for non-normality, so that the cutoff for metacognitive optimality (the use of all available information from the first-order judgment for the second-order judgment) is at 0.

Figure S2 presents such SDT measures for the four difficulty conditions. The top panel presents metacognitive sensitivity, *meta-d′*. Both stimulus mean and variance had a significant influence on metacognitive sensitivity—the higher the mean of the stimulus, the better participants were at discriminating their own correct from their error responses, *F*(1, 19) = 48.4, *p* < .001, η^2^*_p_* = .72, whereas the opposite effect held for stimulus variance, *F*(1, 19) = 34.3, *p* < .001, η^2^*_p_* = .64. These two factors did not interact significantly, *F* < 1. There was no significant difference between the two medium conditions, *t* < 1, *BF_NULL_* = 3.58. Taken together, these findings match the estimates of *A_ROC_* reported in the main text. Here, we aim to compare these findings to differences in first-order performance. With regard to such first-order sensitivity (*d′*; middle panel of Figure S2), there was again a reliable effect of stimulus mean, *F*(1, 19) = 140.0, *p* < .001, η^2^*_p_* = .88, and stimulus variance, *F*(1, 19) = 96.9, *p* < .001, η^2^*_p_* = .84, but no interaction, *F*(1, 19) = 1.0, *p* = .32, η^2^*_p_* = .05.

But does this really mean participants have better metacognitive insight in the easier conditions? Given a metacognitively optimal observer—that is, an observer who uses all available evidence from the first-order decision when making the second-order choice—*meta-d′* reflects the level of evidence that was available from the first order decision to arrive at the judgments of the second-order choice—*d′*. They therefore had to be compared to *d′*, resulting in metacognitive efficiency presented in the bottom panel of Figure S2. For this parameter, there was no reliable influence of neither stimulus mean nor variance, *Fs* < 1, nor was there an interaction between the two factors, *F*(1, 19) = 1.9, *p* = .19, η^2^*_p_* = .09. The two medium conditions were matched, *t* < 1, *BF_NULL_* = 4.16, suggesting that participants were equally good at detecting their own errors in those performance-matched conditions, similar to what we found for *A_ROC_*. Taken together, these results suggest that neither stimulus mean nor variance have an influence on how well participants distinguished between correct and error responses over and above the effect of these factors on the color decision itself.

**References**

Bruyer, R., & Brysbaert, M. (2011). Combining speed and accuracy in cognitive psychology: Is the inverse efficiency score (IES) a better dependent variable than the mean reaction time (RT) and the percentage of errors (PE)? *Psychologica Belgica*, *51*(1), 5–13. doi:10.5334/pb-51-1-5

Fleming, S. M., & Lau, H. C. (2014). How to measure metacognition. *Frontiers in Human Neuroscience*, *8*, 443. doi:10.3389/fnhum.2014.00443

Maniscalco, B., & Lau, H. (2012). A signal detection theoretic approach for estimating metacognitive sensitivity from confidence ratings. *Consciousness and Cognition*, *21*(1), 422–430. doi:10.1016/j.concog.2011.09.021

Table S1

Median split groups

| **Groups** | **Small RT difference** | | | | **Large RT difference** | | | |
| --- | --- | --- | --- | --- | --- | --- | --- | --- |
| **Mean** | **high** | | **low** | | **high** | | **low** | |
| **Variance** | **low** | **high** | **low** | **high** | **low** | **high** | **low** | **high** |
| **Correct RTs (ms)** | 590 | 652 | 647 | 704 | 608 | 708 | 652 | 753 |
| **Error Rate (%)** | 5.0 | 10.7 | 15.4 | 23.8 | 5.5 | 13.2 | 14.5 | 26.0 |
| **Confidence (cor)** | 5.4 | 5.0 | 5.1 | 4.6 | 5.4 | 4.7 | 5.1 | 4.4 |
| **Confidence (err)** | 2.5 | 3.1 | 3.6 | 3.8 | 3.0 | 3.3 | 3.5 | 3.7 |

*Figure S1.* Confidence for the four difficulty color conditions (together for both error and correct trials).

**
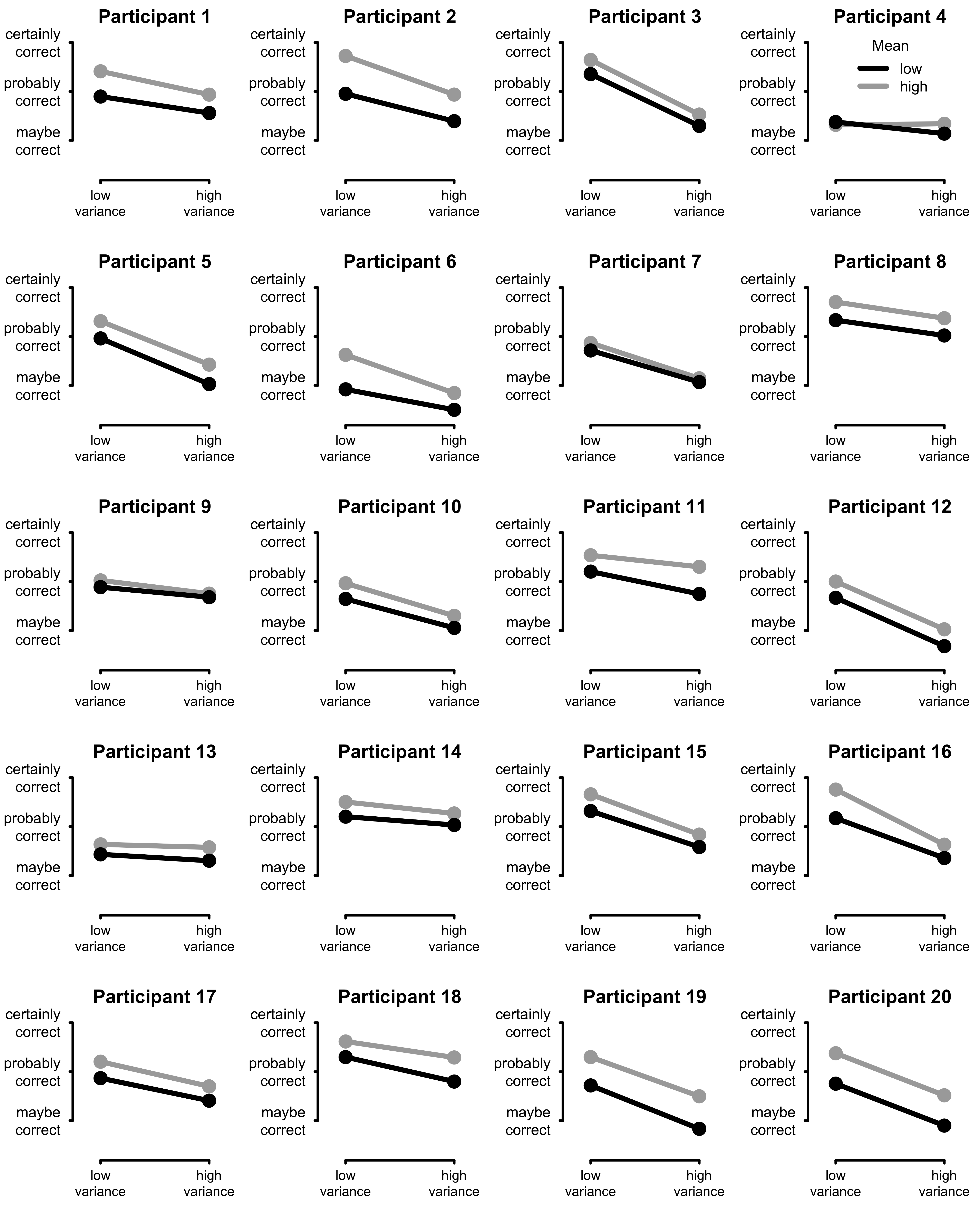
**


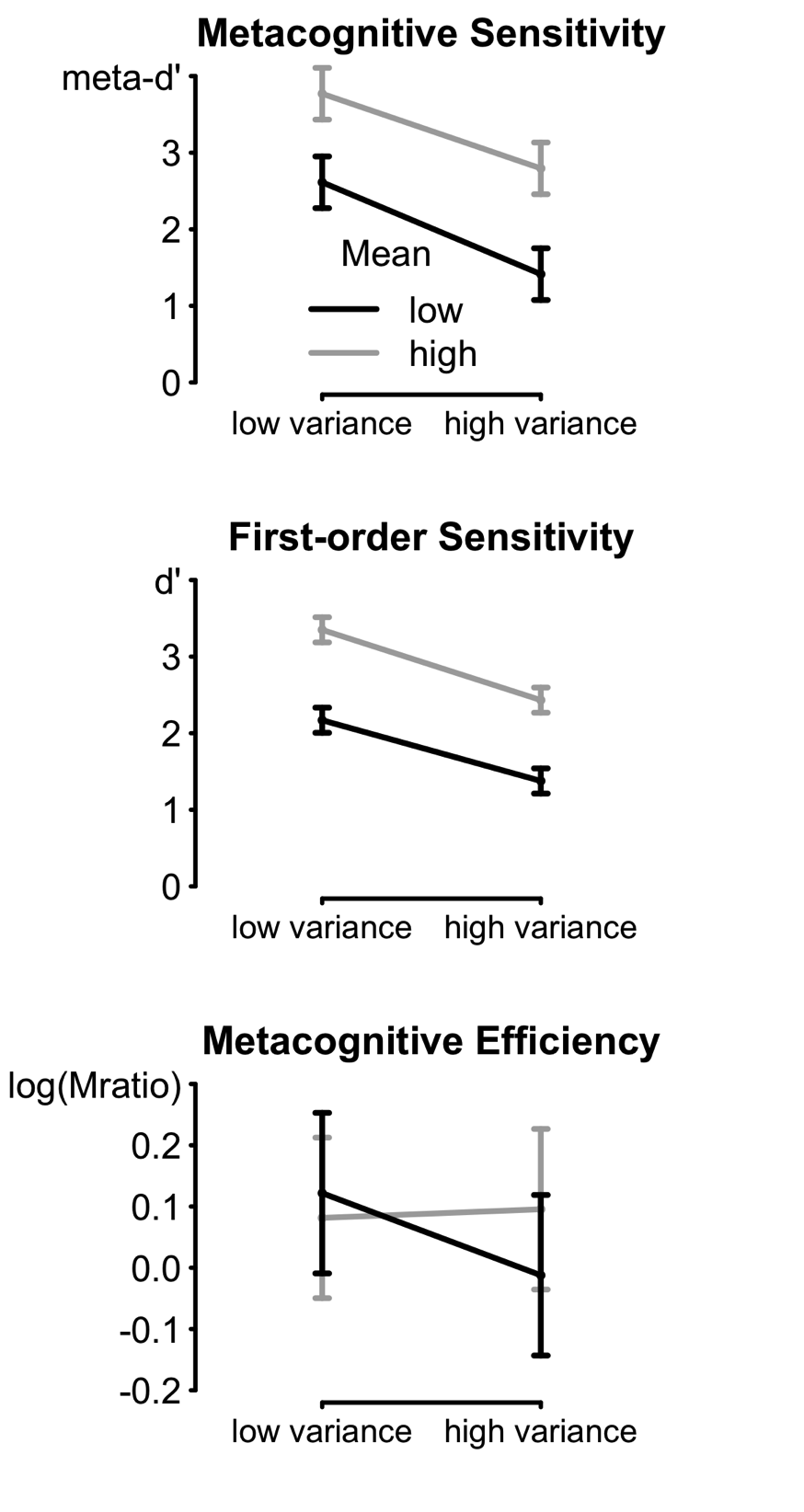
*Figure S2.* Metacognitive sensitivity (*meta-d′*; top panel), first-order sensitivity (*d′*; middle panel), and the common logarithm of metacognitive efficiency (*log(M-ratio)*; bottom panel) as a function of the four difficulty conditions. Both *meta-d′* and *d′* are coded in the same units. Values of *log(M-ratio)* larger than 0 can be interpreted as super-optimal metacognitive behavior.
